# Streaming, Bouncing, and Rotation: The Polka Dance Stimulus

**DOI:** 10.1177/2041669518777259

**Published:** 2018-07-04

**Authors:** Gerard B. Remijn, Tatsuya Yoshizawa, Hiroaki Yano

**Affiliations:** Kyushu University, Fukuoka, Japan; Kanazawa Institute of Technology, Nonoichi, Ishikawa, Japan; Kyushu University, Fukuoka, Japan

**Keywords:** Rivalry or bistability, perceptual organization, visuo-auditory interactions, grouping, spatiotemporal factors

## Abstract

When the objects in a typical stream-bounce stimulus are made to rotate on a circular trajectory, not two but four percepts can be observed: streaming, bouncing, clockwise rotation, and counterclockwise rotation, often with spontaneous reversals between them. When streaming or bouncing is perceived, the objects seem to move on individual, opposite trajectories. When rotation is perceived, however, the objects seem to move in unison on the same circular trajectory, as if constituting the edges of a virtual pane that pivots around its axis. We called this stimulus the Polka Dance stimulus. Experiments showed that with some viewing experience, the viewer can “hold” the rotation percepts. Yet even when doing so, a short sound at the objects’ point of coincidence can induce a bouncing percept. Besides this fast percept switching from rotation to bouncing, an external stimulus might also induce slower rotation direction switches, from clockwise to counterclockwise, or vice versa.

Ambiguous visual stimuli are important tools for studying cognitive processing in general and visual perception in particular. Without undergoing any physical change, ambiguous stimuli can give rise to two or more mutually exclusive percepts, which often alternate (“switch”) spontaneously in the viewer’s mind. Percept formation and switching of ambiguous stimuli have been studied with the focus on viewer traits, such as handedness, gender, and pathological disorders (e.g., see Kornmeier & Bach, 2012), through experimental paradigms that probed early sensory stages of (post)retinal processing to viewer awareness and attentional control.

Here, we introduce a new ambiguous visual stimulus (Movie 1). The stimulus is quadri-stable and proceeds from bistable stream-bounce motion expanded with sinusoidal object rotation. Stream-bounce perception (Metzger, 1934; Michotte, 1946/1963) can be perceived when two identical objects move toward each other, overlap, and then move away from each other. Typically, the objects are seen as moving through each other (“streaming”) or as bouncing off of each other (“bouncing”), in which case each object traverses back to its starting point. Although the streaming percept is often the default, the bouncing percept is facilitated when the two objects decelerate before and accelerate after their point of coincidence with a coincidence speed below 1 deg/s (Zeljko & Grove, 2017), or when a transient stimulus is presented at their point of coincidence (Sekuler, Sekuler, & Lau, 1997). Despite the simplicity of the objects and their physical motion pattern, the perceptual mechanism behind stream-bounce switching is still not clear. Explanations have linked it with bottom-up sensory visual processing, attentional engagement, and probabilistic inference making performed late in the viewer’s processing hierarchy (for a recent discussion see Zeljko & Grove, 2017).

To further study the translation from two-dimensional motion to three-dimensional perception, we added sinusoidal rotation to the stream-bounce stimulus. That is, the two identical objects were made to move continuously over a circular trajectory in a fixed 180-degree relationship with each other. The result is a quadri-stable stimulus that is easy to generate and does not require cross-fixation. As can be seen in Movie 1, the objects can be interpreted as continuously moving on a linear trajectory toward and away from each other, either as streaming or bouncing. However, because of their circular motion while facing each other, the objects can alternatively be seen as constituting the edges of a virtual pane, pivoting around its center point. In this case, the objects can be seen as rotating in unison and, moreover, the viewer can often experience spontaneous switching between clockwise and counterclockwise rotation. Spontaneous reversals in rotation direction are well-documented, for example, in Benussi’s (1917) studies of “Scheinbewegungen” or the silhouette spinner (e.g., Liu et al., 2012).

We called the stimulus the “Polka Dance stimulus” because the movements of the objects (streaming, bouncing, clockwise-, and counterclockwise rotation) resemble those in the dance—or so we assume. Our observations and experiments have shown that naïve viewers do not always report all four percepts spontaneously after 2 minutes of viewing. After explanation of the percepts, however, all viewers do and they often report percept switching. Disambiguating the stimulus can be done by making the objects dissimilar (Movie 2), which basically provides cues that they are moving in three-dimensional space and, hence, are streaming past one another. Alternative percepts, such as waggling or figure-eight movement, have also been reported on occasion.

When asked to “hold” the rotation percept for 5 to 7 physical rotations, viewers (*n*=10) indeed could do so in 86% of the trials. Seeing rotation, clockwise or counterclockwise, implies that the viewer grouped the objects, possibly as the edges of a single virtual pane, or at least as two objects moving in unison. When a sound was presented at the objects’ point of coincidence at the penultimate turn of the 5 to 7 rotations, however, the percentage of bouncing percepts increased significantly from 9% of the trials without sound to 27% of the trials with sound. (Movie 3). This suggests that the objects’ grouping can break down due to the occurrence of a transient, external stimulus presented in a different modality.

While a well-timed single click can facilitate the bouncing percept, rotation percepts can be induced by click-trains. When panned left–right or right–left between headphones, viewers reported a significantly higher incidence of rotation in the Polka Dance stimulus. Finally, informal viewing also suggested that rotation reversals (clockwise to counterclockwise, or vice versa) can be induced by the presentation of an external transient. This relatively slow (<1 second) rotation direction switching due to the presence of a transient stimulus (e.g., Kanai, Moradi, Shimojo, & Verstraten, 2005) contrasts sharply with the fast, almost immediate (<0.1 second) bounce-inducing effect of a transient presented at the objects’ point of coincidence. It seems that both processes can be investigated in tandem by using the Polka Dance stimulus.

**Movie 1.** The Polka Dance stimulus consists of two identical objects, for example of 2 × 0.1 degrees of visual angle, placed above a fixation cross (0.5 × 0.5 degrees). The bars are initially separated by 3 degrees in visual angle and are physically rotated with a fixed distance between them on an imaginary circle, at 3 degrees per second along the circumference.

**Movie 2.** A height difference between the objects in the Polka Dance stimulus facilitates the streaming percept.

**Movie 3.** A click-sound added at the point of the objects' coincidence significantly facilitates the bouncing percept in a Polka Dance stimulus.
